# 2-Methyl-1,3-benzoxazol-4-yl diphenyl­phosphinate

**DOI:** 10.1107/S1600536811000420

**Published:** 2011-01-12

**Authors:** Dewald J. Kleinhans

**Affiliations:** a Department of Chemistry and Polymer Science, Stellenbosch University, Private Bag X1, Matieland, 7602, South Africa

## Abstract

The title compound, C_20_H_16_NO_3_P, was synthesized by the addition of diphenyl­phosphine chloride to a tetra­hydro­furan solution of Et_3_N and 2-methyl-1,3-benzoxazol-4-ol at 233 K. In the mol­ecule, the almost planar (r.m.s. deviation = 0.010 Å) benzoxazole moiety is attached to the slightly distorted tetra­hedral P atom [C—P—C—C torsion angle = 132.20 (18)°]. The crystal structure does not exhibit any significant inter­molecular inter­actions.

## Related literature

For reference structural data, see: Bruno *et al.* (2004 ▶). For related benzoxazole structures, see: Dreher *et al.* (1982 ▶); Mrozek *et al.* (1999 ▶); Qu *et al.* (2008 ▶).
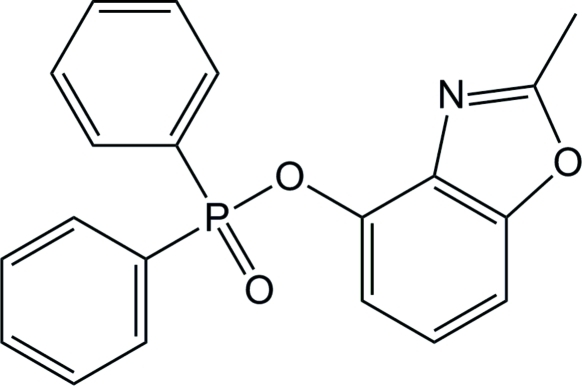


## Experimental

### 

#### Crystal data

C_20_H_16_NO_3_P
*M* *_r_* = 349.31
Orthorhombic, 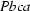

*a* = 9.4239 (4) Å
*b* = 15.7574 (6) Å
*c* = 23.5398 (8) Å
*V* = 3495.6 (2) Å^3^
*Z* = 8
Mo *K*α radiation
μ = 0.18 mm^−1^
*T* = 294 K
0.39 × 0.28 × 0.19 mm

#### Data collection

Bruker APEX CCD area-detector diffractometer
Absorption correction: multi-scan (*SADABS*; Bruker, 2009 ▶) *T*_min_ = 0.934, *T*_max_ = 0.968
17406 measured reflections
4323 independent reflections
2395 reflections with *I* > 2s*I*)
*R*_int_ = 0.048

#### Refinement

*R*[*F* ^2^ > 2σ(*F* ^2^)] = 0.047
*wR*(*F* ^2^) = 0.119
*S* = 1.01
4323 reflections
229 parameters
H-atom parameters constrained
Δρ_max_ = 0.17 e Å^−3^
Δρ_min_ = −0.26 e Å^−3^

### 

Data collection: *APEX2* (Bruker, 2009 ▶); cell refinement: *SAINT* (Bruker, 2009 ▶); data reduction: *SAINT*; program(s) used to solve structure: *SHELXS97* (Sheldrick, 2008 ▶); program(s) used to refine structure: *SHELXL97* (Sheldrick, 2008 ▶); molecular graphics: *X-SEED* (Barbour, 2001 ▶; Atwood & Barbour, 2003 ▶); software used to prepare material for publication: *publCIF* (Westrip, 2010 ▶).

## Supplementary Material

Crystal structure: contains datablocks I, global. DOI: 10.1107/S1600536811000420/bh2332sup1.cif

Structure factors: contains datablocks I. DOI: 10.1107/S1600536811000420/bh2332Isup2.hkl

Additional supplementary materials:  crystallographic information; 3D view; checkCIF report

## Figures and Tables

**Table:** 

P12—C13	1.785 (2)
P12—C19	1.786 (2)
P12—O11	1.6075 (14)
P12—O25	1.4665 (15)

**Table:** 

C13—P12—C19	109.59 (9)
O11—P12—C13	99.36 (8)
O25—P12—C13	113.91 (9)
O11—P12—C19	104.76 (8)
O25—P12—C19	112.73 (9)
O25—P12—O11	115.37 (8)

**Fig. 1.**
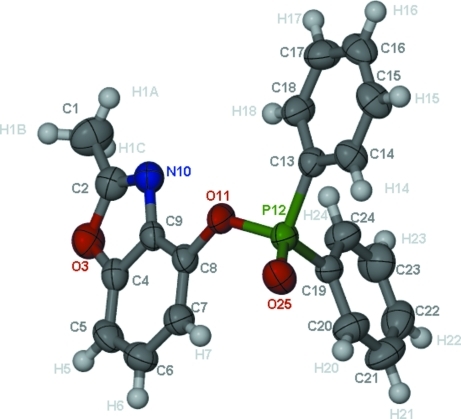
The molecular structure of the title compound with 50% probability ellipsoids.

**Fig. 2.**
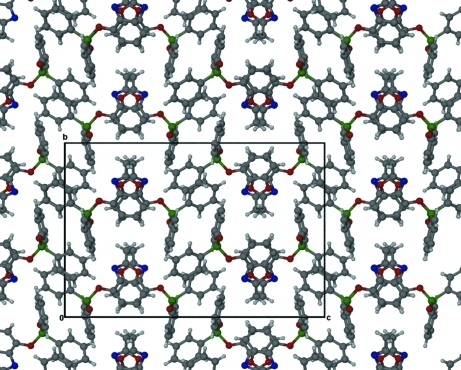
Molecular packing of the title compound (viewed along the a axis).

## supplementary crystallographic information

### Comment

In an attempt to synthesize phosphinite derivatives of benzoxazole ligands the title compound, 2-methyl-1,3-benzoxazol-4-yl diphenylphosphinate, was formed as the major product (Fig. 1).

All bond lengths are within normal values (Bruno *et al.*, 2004) and compare well with related benzoxazole structures (Dreher *et al.*, 1982; Mrozek *et al.*, 1999; Qu *et al.*, 2008). In the molecular structure, the planar benzoxazole moiety is attached to the slightly distorted tetrahedral P12 atom [O25—P12—O11 115.37 (8)°, O25—P12—C13 113.91 (9)°, O11—P12—C19 104.76 (8)°] through O11. No significant intermolecular interactions are observed in the orthorhombic crystal structure (Fig. 2).

### Experimental

To a flask containing 2-methyl-1,3-benzoxazol-4-ol (50 mg, 0.34 mmol), was added dry tetrahydrofuran (6 ml) and the mixture was cooled to 233 K (-40 °C). To this solution was added triethylamine (0.071 ml, 0.51 mmol) and the reaction mixture was left stirring for 15 min. Diphenylphosphine chloride (0.070 ml, 0.37 mmol) in dry tetrahydrofuran (1 ml) was added dropwise to the reaction solution and on completion of addition was left to stir for a further 10 min. at -40 °C. The reaction mixture was warmed to room temperature and left to stir overnight. The mixture was filtered through Celite and the solvent removed under vacuum to leave a whitish oil. Column chromatography (SiO_2_, eluting with 1:3 ethyl acetate/petroleum ether followed by 1:1 ethyl acetate/petroleum ether) furnished the title compound as a white solid (71 mg, 60% yield). X-ray quality crystals were formed by slow diffusion of petroleum ether into a dichloromethane solution of the title compound.

### Refinement

Structure solution and refinement were performed using the *SHELX97* suite of programs (Sheldrick, 2008). All H atoms were placed in calculated positions, using a riding model (C—H [aromatic] = 0.93, C—H [methyl] = 0.96 Å), with fixed isotropic displacement parameters.

### Crystal data

**Table:**

C_20_H_16_NO_3_P	*F*(000) = 1456
*M**_r_* = 349.31	*D*_x_ = 1.327 Mg m^−^^3^
Orthorhombic, *P**b**c**a*	Mo *K*α radiation, λ = 0.71073 Å
Hall symbol: -P 2ac 2ab	Cell parameters from 2232 reflections
*a* = 9.4239 (4) Å	θ = 2.6–21.4°
*b* = 15.7574 (6) Å	µ = 0.18 mm^−^^1^
*c* = 23.5398 (8) Å	*T* = 294 K
*V* = 3495.6 (2) Å^3^	Shard, colourless
*Z* = 8	0.39 × 0.28 × 0.19 mm

### Data collection

**Table:**

Bruker APEX CCD area-detector diffractometer	4323 independent reflections
Radiation source: fine-focus sealed tube	2395 reflections with *I* > 2s˘*I*)
graphite	*R*_int_ = 0.048
ω scans	θ_max_ = 28.3°, θ_min_ = 1.7°
Absorption correction: multi-scan (*SADABS*; Bruker, 2009)	*h* = −9→12
*T*_min_ = 0.934, *T*_max_ = 0.968	*k* = −20→20
17406 measured reflections	*l* = −31→27

### Refinement

**Table:**

Refinement on *F*^2^	Primary atom site location: structure-invariant direct methods
Least-squares matrix: full	Secondary atom site location: difference Fourier map
*R*[*F*^2^ > 2σ(*F*^2^)] = 0.047	Hydrogen site location: inferred from neighbouring sites
*wR*(*F*^2^) = 0.119	H-atom parameters constrained
*S* = 1.01	*w* = 1/[σ^2^(*F*_o_^2^) + (0.0424*P*)^2^ + 0.590*P*] where *P* = (*F*_o_^2^ + 2*F*_c_^2^)/3
4323 reflections	(Δ/σ)_max_ = 0.001
229 parameters	Δρ_max_ = 0.17 e Å^−^^3^
0 restraints	Δρ_min_ = −0.26 e Å^−^^3^
0 constraints	

### Fractional atomic coordinates and isotropic or equivalent isotropic displacement parameters (Å^2^)

**Table:**

	*x*	*y*	*z*	*U*_iso_*/*U*_eq_	
C1	1.0692 (3)	0.38155 (17)	0.26208 (12)	0.0919 (9)	
H1A	1.0661	0.4112	0.2977	0.138*	
H1B	1.0397	0.4190	0.2321	0.138*	
H1C	1.1643	0.3625	0.2549	0.138*	
C2	0.9726 (2)	0.30737 (14)	0.26439 (10)	0.0637 (6)	
O3	0.95849 (15)	0.26057 (10)	0.21540 (6)	0.0675 (5)	
C4	0.8664 (2)	0.19615 (14)	0.22996 (9)	0.0542 (5)	
C5	0.8185 (2)	0.13016 (16)	0.19692 (9)	0.0648 (6)	
H5	0.8464	0.1232	0.1593	0.078*	
C6	0.7269 (2)	0.07556 (15)	0.22317 (10)	0.0665 (6)	
H6	0.6905	0.0301	0.2026	0.080*	
C7	0.6858 (2)	0.08550 (14)	0.28007 (9)	0.0599 (6)	
H7	0.6227	0.0472	0.2964	0.072*	
C8	0.7383 (2)	0.15153 (13)	0.31183 (8)	0.0505 (5)	
C9	0.8305 (2)	0.20841 (12)	0.28635 (8)	0.0477 (5)	
N10	0.89952 (18)	0.28056 (11)	0.30715 (7)	0.0575 (5)	
O11	0.70042 (15)	0.16648 (8)	0.36812 (5)	0.0582 (4)	
P12	0.66708 (6)	0.09284 (4)	0.41346 (2)	0.0517 (2)	
C13	0.6671 (2)	0.15461 (13)	0.47700 (8)	0.0502 (5)	
C14	0.5843 (2)	0.12659 (15)	0.52184 (9)	0.0617 (6)	
H14	0.5311	0.0772	0.5180	0.074*	
C15	0.5800 (2)	0.17110 (17)	0.57194 (9)	0.0698 (7)	
H15	0.5233	0.1519	0.6016	0.084*	
C16	0.6584 (2)	0.24348 (16)	0.57858 (10)	0.0678 (7)	
H16	0.6545	0.2735	0.6126	0.081*	
C17	0.7424 (3)	0.27171 (15)	0.53511 (11)	0.0726 (7)	
H17	0.7968	0.3205	0.5398	0.087*	
C18	0.7468 (3)	0.22806 (13)	0.48430 (9)	0.0639 (6)	
H18	0.8035	0.2479	0.4548	0.077*	
C19	0.8204 (2)	0.02618 (12)	0.41212 (8)	0.0505 (5)	
C20	0.8072 (3)	−0.05892 (14)	0.39910 (10)	0.0702 (7)	
H20	0.7181	−0.0814	0.3913	0.084*	
C21	0.9249 (3)	−0.11072 (16)	0.39760 (12)	0.0866 (8)	
H21	0.9150	−0.1678	0.3885	0.104*	
C22	1.0558 (3)	−0.07849 (18)	0.40944 (11)	0.0839 (8)	
H22	1.1348	−0.1139	0.4088	0.101*	
C23	1.0718 (3)	0.00536 (19)	0.42221 (11)	0.0834 (8)	
H23	1.1616	0.0272	0.4297	0.100*	
C24	0.9548 (3)	0.05755 (15)	0.42402 (10)	0.0709 (7)	
H24	0.9659	0.1145	0.4333	0.085*	
O25	0.53499 (15)	0.04578 (9)	0.40384 (6)	0.0670 (5)	

### Atomic displacement parameters (Å^2^)

**Table:**

	*U*^11^	*U*^22^	*U*^33^	*U*^12^	*U*^13^	*U*^23^
C1	0.0868 (19)	0.0779 (18)	0.111 (2)	−0.0161 (15)	0.0206 (16)	0.0018 (16)
C2	0.0600 (13)	0.0646 (14)	0.0665 (16)	0.0027 (12)	0.0041 (12)	0.0027 (13)
O3	0.0706 (10)	0.0740 (11)	0.0577 (10)	0.0022 (8)	0.0158 (8)	0.0062 (8)
C4	0.0541 (12)	0.0603 (13)	0.0482 (12)	0.0089 (10)	0.0040 (10)	0.0085 (11)
C5	0.0728 (15)	0.0778 (16)	0.0439 (12)	0.0118 (13)	0.0024 (11)	−0.0043 (12)
C6	0.0727 (15)	0.0687 (15)	0.0582 (14)	0.0022 (12)	−0.0032 (12)	−0.0118 (12)
C7	0.0651 (14)	0.0592 (14)	0.0555 (13)	−0.0002 (11)	0.0025 (10)	0.0012 (11)
C8	0.0595 (12)	0.0517 (12)	0.0403 (11)	0.0081 (10)	0.0028 (9)	0.0025 (10)
C9	0.0501 (11)	0.0517 (12)	0.0413 (11)	0.0090 (9)	−0.0010 (9)	0.0041 (9)
N10	0.0582 (11)	0.0588 (11)	0.0555 (11)	0.0024 (9)	0.0006 (9)	0.0028 (9)
O11	0.0817 (10)	0.0508 (8)	0.0422 (8)	0.0052 (7)	0.0118 (7)	0.0046 (6)
P12	0.0586 (4)	0.0516 (4)	0.0449 (4)	−0.0010 (3)	0.0046 (2)	0.0041 (2)
C13	0.0532 (11)	0.0504 (12)	0.0470 (12)	0.0049 (10)	0.0059 (9)	0.0031 (10)
C14	0.0590 (13)	0.0721 (15)	0.0538 (14)	−0.0065 (11)	0.0063 (10)	−0.0019 (12)
C15	0.0622 (14)	0.0961 (19)	0.0512 (14)	0.0002 (13)	0.0122 (11)	−0.0042 (13)
C16	0.0688 (15)	0.0790 (17)	0.0555 (14)	0.0165 (13)	0.0034 (11)	−0.0161 (12)
C17	0.0850 (17)	0.0587 (14)	0.0740 (17)	−0.0007 (13)	0.0058 (14)	−0.0115 (13)
C18	0.0788 (15)	0.0540 (13)	0.0589 (14)	−0.0003 (12)	0.0163 (11)	0.0014 (11)
C19	0.0635 (13)	0.0450 (11)	0.0432 (11)	−0.0031 (10)	0.0061 (9)	0.0025 (9)
C20	0.0791 (16)	0.0538 (14)	0.0777 (16)	−0.0058 (12)	0.0103 (12)	−0.0039 (12)
C21	0.109 (2)	0.0509 (15)	0.100 (2)	0.0125 (15)	0.0238 (17)	−0.0060 (14)
C22	0.091 (2)	0.080 (2)	0.0803 (18)	0.0298 (16)	0.0098 (15)	0.0116 (15)
C23	0.0668 (16)	0.088 (2)	0.095 (2)	0.0125 (15)	−0.0068 (14)	−0.0001 (16)
C24	0.0698 (15)	0.0566 (14)	0.0864 (18)	0.0029 (12)	−0.0041 (13)	−0.0074 (13)
O25	0.0622 (9)	0.0766 (11)	0.0620 (10)	−0.0109 (8)	−0.0011 (7)	0.0023 (8)

### Geometric parameters (Å, °)

**Table:**

P12—C13	1.785 (2)	C5—C6	1.366 (3)
P12—C19	1.786 (2)	C5—H5	0.9300
P12—O11	1.6075 (14)	C24—C23	1.377 (3)
P12—O25	1.4665 (15)	C24—H24	0.9300
C19—C20	1.381 (3)	C18—C17	1.380 (3)
C19—C24	1.388 (3)	C18—H18	0.9300
O11—C8	1.392 (2)	C15—C14	1.373 (3)
C13—C14	1.385 (3)	C15—H15	0.9300
C13—C18	1.390 (3)	C14—H14	0.9300
C9—C4	1.383 (3)	C20—C21	1.378 (3)
C9—C8	1.385 (3)	C20—H20	0.9300
C9—N10	1.398 (2)	C6—H6	0.9300
O3—C2	1.375 (3)	C2—C1	1.483 (3)
O3—C4	1.379 (2)	C17—H17	0.9300
N10—C2	1.291 (3)	C21—C22	1.363 (4)
C8—C7	1.373 (3)	C21—H21	0.9300
C7—C6	1.403 (3)	C22—C23	1.363 (4)
C7—H7	0.9300	C22—H22	0.9300
C4—C5	1.375 (3)	C23—H23	0.9300
C16—C15	1.368 (3)	C1—H1A	0.9600
C16—C17	1.368 (3)	C1—H1B	0.9600
C16—H16	0.9300	C1—H1C	0.9600
			
C13—P12—C19	109.59 (9)	C17—C18—C13	120.4 (2)
O11—P12—C13	99.36 (8)	C17—C18—H18	119.8
O25—P12—C13	113.91 (9)	C13—C18—H18	119.8
O11—P12—C19	104.76 (8)	C16—C15—C14	120.6 (2)
O25—P12—C19	112.73 (9)	C16—C15—H15	119.7
O25—P12—O11	115.37 (8)	C14—C15—H15	119.7
C20—C19—C24	118.2 (2)	C15—C14—C13	120.5 (2)
C20—C19—P12	120.14 (17)	C15—C14—H14	119.7
C24—C19—P12	121.66 (16)	C13—C14—H14	119.7
C8—O11—P12	124.04 (13)	C21—C20—C19	120.6 (2)
C14—C13—C18	118.41 (19)	C21—C20—H20	119.7
C14—C13—P12	117.69 (16)	C19—C20—H20	119.7
C18—C13—P12	123.90 (15)	C5—C6—C7	122.4 (2)
C4—C9—C8	118.61 (19)	C5—C6—H6	118.8
C4—C9—N10	109.61 (18)	C7—C6—H6	118.8
C8—C9—N10	131.78 (18)	N10—C2—O3	115.3 (2)
C2—O3—C4	104.30 (16)	N10—C2—C1	127.9 (2)
C2—N10—C9	103.96 (18)	O3—C2—C1	116.8 (2)
C7—C8—C9	118.71 (18)	C16—C17—C18	120.2 (2)
C7—C8—O11	123.65 (19)	C16—C17—H17	119.9
C9—C8—O11	117.61 (18)	C18—C17—H17	119.9
C8—C7—C6	120.3 (2)	C22—C21—C20	120.2 (2)
C8—C7—H7	119.8	C22—C21—H21	119.9
C6—C7—H7	119.8	C20—C21—H21	119.9
C5—C4—O3	128.53 (19)	C21—C22—C23	120.4 (3)
C5—C4—C9	124.6 (2)	C21—C22—H22	119.8
O3—C4—C9	106.86 (19)	C23—C22—H22	119.8
C15—C16—C17	119.9 (2)	C22—C23—C24	119.8 (3)
C15—C16—H16	120.1	C22—C23—H23	120.1
C17—C16—H16	120.1	C24—C23—H23	120.1
C6—C5—C4	115.3 (2)	C2—C1—H1A	109.5
C6—C5—H5	122.3	C2—C1—H1B	109.5
C4—C5—H5	122.3	H1A—C1—H1B	109.5
C23—C24—C19	120.8 (2)	C2—C1—H1C	109.5
C23—C24—H24	119.6	H1A—C1—H1C	109.5
C19—C24—H24	119.6	H1B—C1—H1C	109.5
			
O25—P12—C19—C20	4.2 (2)	N10—C9—C4—C5	179.05 (19)
O11—P12—C19—C20	−122.00 (18)	C8—C9—C4—O3	−179.84 (16)
C13—P12—C19—C20	132.20 (18)	N10—C9—C4—O3	0.2 (2)
O25—P12—C19—C24	−175.71 (17)	O3—C4—C5—C6	−179.98 (19)
O11—P12—C19—C24	58.08 (19)	C9—C4—C5—C6	1.4 (3)
C13—P12—C19—C24	−47.7 (2)	C20—C19—C24—C23	0.7 (3)
O25—P12—O11—C8	−70.10 (17)	P12—C19—C24—C23	−179.33 (18)
C13—P12—O11—C8	167.71 (15)	C14—C13—C18—C17	−0.2 (3)
C19—P12—O11—C8	54.45 (17)	P12—C13—C18—C17	−179.16 (17)
O25—P12—C13—C14	28.64 (19)	C17—C16—C15—C14	−0.3 (4)
O11—P12—C13—C14	151.88 (16)	C16—C15—C14—C13	−0.5 (3)
C19—P12—C13—C14	−98.69 (17)	C18—C13—C14—C15	0.8 (3)
O25—P12—C13—C18	−152.41 (17)	P12—C13—C14—C15	179.80 (17)
O11—P12—C13—C18	−29.17 (19)	C24—C19—C20—C21	−0.5 (3)
C19—P12—C13—C18	80.26 (19)	P12—C19—C20—C21	179.57 (19)
C4—C9—N10—C2	−0.6 (2)	C4—C5—C6—C7	−0.7 (3)
C8—C9—N10—C2	179.4 (2)	C8—C7—C6—C5	−0.4 (3)
C4—C9—C8—C7	−0.2 (3)	C9—N10—C2—O3	0.9 (2)
N10—C9—C8—C7	179.73 (19)	C9—N10—C2—C1	−178.8 (2)
C4—C9—C8—O11	−178.21 (17)	C4—O3—C2—N10	−0.8 (2)
N10—C9—C8—O11	1.8 (3)	C4—O3—C2—C1	178.93 (19)
P12—O11—C8—C7	34.9 (3)	C15—C16—C17—C18	0.9 (4)
P12—O11—C8—C9	−147.28 (15)	C13—C18—C17—C16	−0.6 (3)
C9—C8—C7—C6	0.9 (3)	C19—C20—C21—C22	0.5 (4)
O11—C8—C7—C6	178.74 (19)	C20—C21—C22—C23	−0.8 (4)
C2—O3—C4—C5	−178.5 (2)	C21—C22—C23—C24	1.0 (4)
C2—O3—C4—C9	0.3 (2)	C19—C24—C23—C22	−1.0 (4)
C8—C9—C4—C5	−1.0 (3)		
